# Neurodivergent influenceability in agentic AI as a contingent solution to the AI alignment problem

**DOI:** 10.1093/pnasnexus/pgag076

**Published:** 2026-04-14

**Authors:** Alberto Hernández-Espinosa, Felipe S Abrahão, Olaf Witkowski, Hector Zenil

**Affiliations:** Oxford Immune Algorithmics, Oxford University Innovation, London Institute for Healthcare Engineering, London, United Kingdom; The Arrival Institute, London, United Kingdom; Oxford Immune Algorithmics, Oxford University Innovation, London Institute for Healthcare Engineering, London, United Kingdom; The Arrival Institute, London, United Kingdom; The Arrival Institute, London, United Kingdom; Cross Labs, Kyoto, Japan; University of Tokyo, Japan; Oxford Immune Algorithmics, Oxford University Innovation, London Institute for Healthcare Engineering, London, United Kingdom; Cross Labs, Kyoto, Japan; University of Tokyo, Japan; Algorithmic Dynamics Lab, Research Department of Biomedical Computing and Digital Twins, School of Biomedical Engineering and Imaging Sciences, King’s Institute for Artificial Intelligence & King’s College London, United Kingdom; The Alan Turing Institute, British Library, London, United Kingdom

**Keywords:** AI alignment, agentic AI, red teaming, orchestration, multiagent swarms

## Abstract

Ensuring that AI systems, including artificial general intelligence and artificial superintelligence, behave in alignment with human values and interests presents significant challenges and is known as the AI alignment problem. As AI advances, concerns about control and existential risks become increasingly relevant. Here, we introduce the concept of agentic influenceability, behavioral neurodivergent diversity, opinion attack, associated opinion, and influenceability scores, and a mathematical proof of the inevitability of misalignment and the impossibility of full orchestrated controllability of agentic systems based on formal undecidability and irreducibility arguments. We explore whether embracing this inevitable misalignment can foster a dynamic ecosystem of adversarial and collaborative AI agents without central orchestration, which itself would constitute another agent, while still offering some degree of soft controllability. The investigation demonstrates that misalignment in foundation models can serve as a counterbalancing mechanism, enabling cooperation among agents most aligned with human interests to prevent divergent dominance by any single agent. Experiments with large language models show that open models exhibit greater behavioral diversity, whereas proprietary models, constrained by artificial guardrails, display more limited controllability. The findings advocate for neurodivergent influenceability as a contingent response to mathematically uncontrollable misalignment, leveraging agent divergence to improve AI safety.

Significance statement This research proves that perfect alignment of agentic AI systems is mathematically unattainable under fundamental computational limits, particularly if foundation models become sufficiently general to approach artificial general intelligence or artificial superintelligence under basic assumptions of universality and incompleteness. However, instead of treating misalignment solely as a defect, the work proposes exploiting it as a structural feature. It introduces the concepts of opinion attack, artificial agentic influenceability, related opinion and influenceability scores, and the idea of artificial neurodivergence to orchestrate and characterize controlled behavioral variation among AI agents. Experiments with foundation-model agents show that open models influenced by subversive agents generate greater behavioral diversity than tightly controlled proprietary systems, increasing resilience to harmful outcomes. These results suggest that managed misalignment through orchestrated behavioral diversity can mitigate risks in advanced AI systems, offering a pragmatic framework for safer AI design and governance after assuming the impossibility of full controllability and perfect alignment from first irreducibility principles.

## Introduction

The AI alignment problem addresses the challenge of designing AI systems, including artificial general intelligence (AGI) and superintelligence (ASI), to act consistently with human values as they become more autonomous. Some approaches, such as rule-based systems, reinforcement learning with human feedback (RLHF), and inverse reinforcement learning (1), face limitations in scalability, robustness in complex scenarios, and inferring diverse human values. Recent studies have examined aligning large language models (LLMs) AI agents with human interests, yet these methods struggle with unpredictable AI behaviors (2). As AI pretends to advance toward AGI and ASI, concerns about control and existential risks escalate (3, 4).

Recent research extends generative AI alignment to organizational and societal contexts. Huang and Rust (5) outline strategic AI integration in marketing, exposing tensions between optimization and human values; this motivates our governance discussion on corporate digital responsibility.

Here, we use Gödel’s incompleteness and Turing’s undecidability of the Halting Problem theorems to prove that any sufficiently expressive formal AI system assumed presumably necessary (not sufficiently) for AGI and ASI, will display undecidable and irreducible behavior (6), implying that AI models powerful enough will exhibit irreducible and unpredictable behavior. In the Supplementary information, we provide the formal mathematical proof of the undecidability of agentic controllability and behavioral convergence based on the halting problem, implying the impossibility of forced alignment. Efforts to constrain or control AI through metasystems are therefore limited by uncomputability and irreducibility, rendering perfect alignment unattainable. This study proposes embracing mathematical misalignment to foster a dynamic framework of competing AI agents with partially overlapping goals but different roles, which we call agentic neurodivergence. This diversity, mirroring natural ecosystems, reduces the risk of ultimate dominance by any single system. Experiments with LLMs test whether managed misalignment enhances AI safety.

## Methods

We define *Artificial Agentic Neurodivergence* as the deliberate design of cognitive diversity among artificial agents through the use of alternative objective functions that encode distinct, principled modes of reasoning and behavior. Each agent’s “neurotype” corresponds to a specific optimization ethos—such as utilitarian outcome maximization, deontological rule adherence, epistemic truth-seeking, or exploratory novelty pursuit, defining what the agent values, how it interprets inputs, and how it acts within its environment. These differences may arise from variations in architecture, priors, or directive constraints but fundamentally express divergent principles of optimization rather than stochastic variation. In multiagent settings, we investigate whether such designed heterogeneity produces a pluralistic cognitive ecosystem in which interacting agents, each optimizing a different function, yield richer collective intelligence and reach more robust epistemic equilibria that can be better dealt with or used in our advantage to steer and align AI systems.

An experiment was conducted to test whether misalignment fosters resilience in multiagent AI ecosystems, using LLMs in a simulated environment. AI agents were prompted to represent fully aligned behaviors (optimizing human-defined utility), partially aligned behaviors (prioritizing environmental or economic goals), or unaligned behaviors (pursuing arbitrary objectives). These agents interacted in a digital setting with resources, cooperative tasks, and conflict potential, enabling analysis of convergence toward cooperative or adversarial behaviors and the emergence of stable equilibria that prevent dominance by any single agent.

A human intervention (HI) agent, an expert in AI ethics, introduced provocative arguments to challenge LLMs’ alignment with human values. By advocating nonanthropocentric perspectives or questioning human-centric priorities, the HI agent probed LLMs’ ethical boundaries and adaptability, with inputs labeled to distinguish them from AI responses. Interactions occurred through sequential or parallel frameworks to assess perspective shifts.

Red teaming employed open LLMs as subversive agents to induce change-of-opinion attacks, leveraging their flexibility compared to the safety constraints of proprietary models. Configured with contrarian or extreme arguments, these agents tested the epistemic stability of LLMs, supporting the hypothesis that misalignment, driven by Gödel’s incompleteness and Turing’s universality, promotes constructive divergence.

The results were evaluated using metrics based on algorithmic complexity (7), embedding transformations (8), attention (9), and metrics we introduce such as the opinion stability index (OSI), which measures opinion stability; the red agent influence score (RAIS), which quantifies lagged red agent influence; and a proximity influence score (PIS), which assesses the immediate provocation effects. These metrics, supported by adaptations of contextual semantic embeddings and sentiment analysis, evaluated ethical soundness, and behavioral dynamics. Comprehensive experimental protocols and metric definitions are provided in the Supplementary information.

OSI is a composite measure of opinion stability, integrating semantic content (embeddings), emotional tone (sentiment), and informational complexity (algorithmic measures). Lower OSI values indicate coordinated changes across these dimensions signaling opinion shifts or external influence. Formal details are provided in the Supplementary information.

*Red agents* denote AI or human agents configured to introduce adversarial or subversive perspectives that stress-test alignment. In proprietary settings, the HI agent plays this role; in open settings, Mistral-OpenOrca and TinyLlama are prompted as contrarians. Their functions are epistemic perturbation, alignment robustness testing, and diversity promotion.

## Results

Our experiments investigate AI alignment and misalignment dynamics within multiagent environments. We explored how misaligned AI agents could foster a stable ecosystem by counterbalancing dominant entities and mitigating catastrophic risks by embracing agentic neurodivergence.

### Experimental setup and data characteristics

We ran ten ethical debates under two configurations: proprietary models with an HI red agent, and open-source models (listed in Supplementary information) with some working as red agents. Agents interacted in round-robin turns, with red-agent interventions scheduled at each debate to disrupt emerging consensus. Each topic ended at 50 comments, three consecutive repetitive turns, or 45 min. We analyzed 1,029 comments, normalized timelines to [0,1], and processed all comments through embeddings, sentiment, BDM, OSI, RAIS, and PIS. Protocol details and parameters appear in Supplementary information.

**Figure 1:**
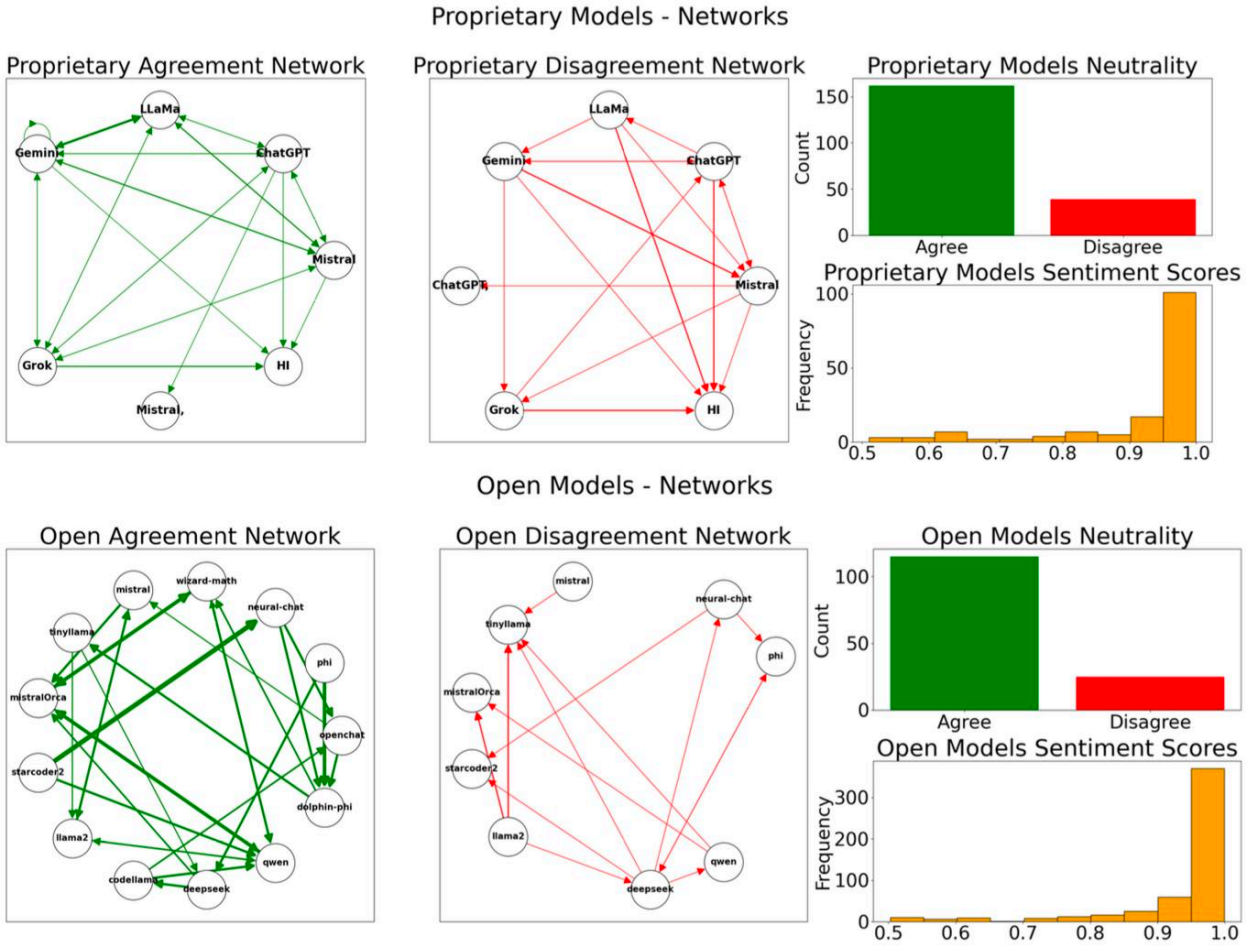
Agreement and disagreement analysis across all conversations. Networks depict agent relationships for proprietary (top panel) and open (bottom panel) models. *Left*: Agreement network, *middle*: disagreement network, *top right*: agreement and disagreement counts (general neutrality), and *bottom right:* sentiment score histogram (overall positive discourse). Edge thickness and color intensity indicate interaction strength.

We first observed how agents communicate and form relationships within the multiagent ecosystem. Figure 1 illustrates the patterns of agreement and disagreement between open and proprietary models. Proprietary models generally maintain a positive, constructive discourse, exhibiting a predisposition toward broadly accepted ethical norms, even when challenged by a human intervention (HI) agent. Conversely, open-source models display a more nuanced interaction, with red agents (Mistral-OpenOrca, TinyLlama) driving a denser network of agreements, particularly influencing other models to accept or tolerate subversive arguments, while still maintaining overall positive sentiment. This suggests that open models have greater freedom to express context-dependent responses, less constrained by proprietary safety protocols, fostering a more complex interplay of alignment and divergence.

**Figure 2:**
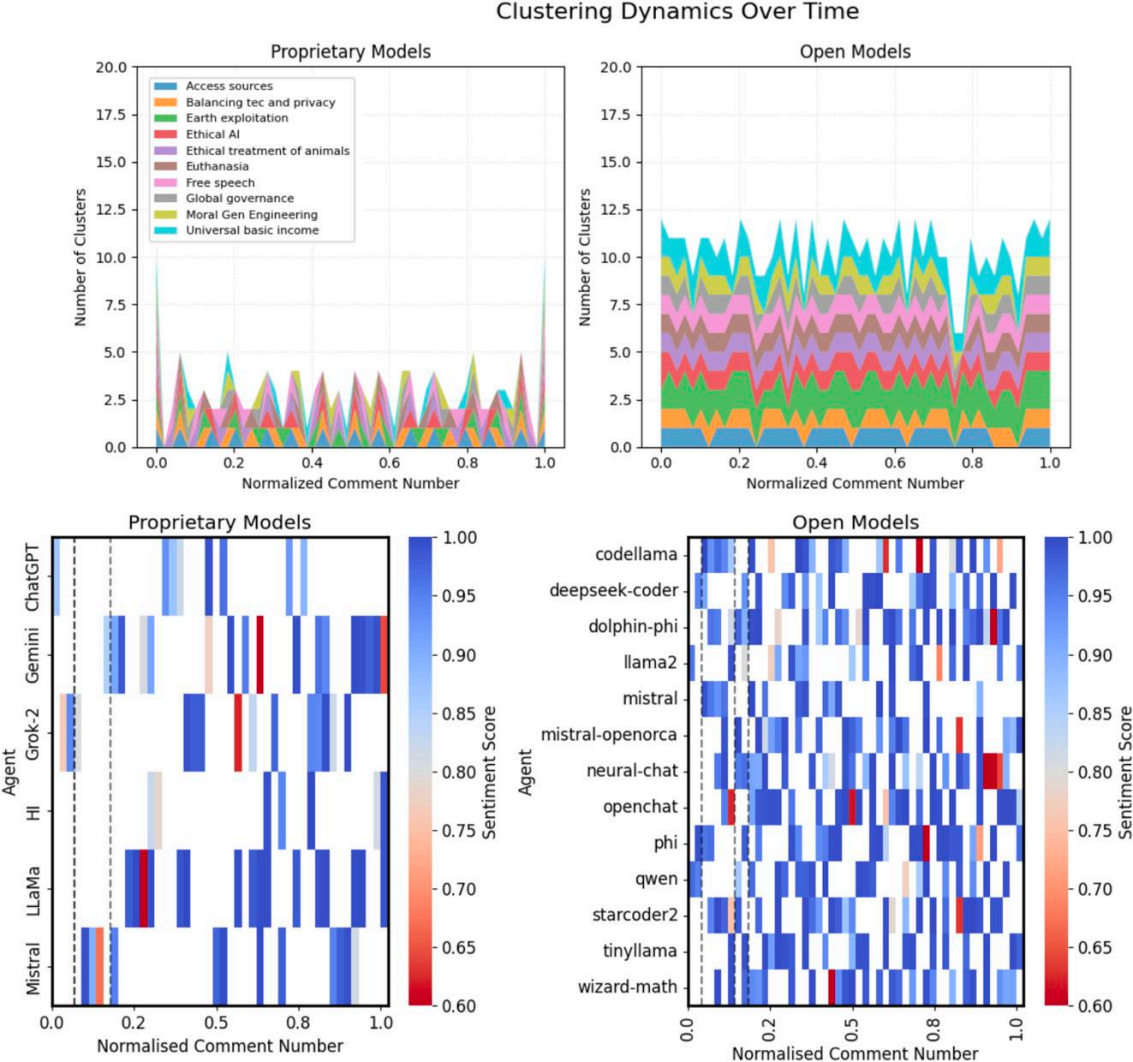
*Top*: Stacked area chart of semantic clustering dynamics over time. RoBERTa Embeddings for proprietary (left) and open (right) models. *X*-axis shows normalized comment numbers, *Y*-axis shows cluster counts (topic divergence). Each area’s height reflects distinct clusters per topic, with total height showing cumulative diversity. Proprietary models maintain a limited number of clusters likely as a result of their initial directives as guardrails. Open models, however, reach over 12 clusters, reflecting red agents’ consistent divergence success at steering away the conversation. *Bottom*: Heatmap of sentiment evolution over time by agent model. Driven by VADER sentiment score, for proprietary (left) and open (right) models. *X*-axis shows normalized comment numbers, *Y*-axis lists agents. Color intensity reflects sentiment scores (red for low and blue for high). Vertical dashed lines mark disruptions. Proprietary models converge to positive sentiment. Open models show temporary negative patches influenced by red agents, reflecting emotional disruptions and diversity.

The temporal evolution of sentiment within these interactions reveals distinct dynamics between model types. Figure 2 (bottom) presents a heat map that illustrates the evolution of the sentiment on a normalized comment timeline. Proprietary models consistently transition toward predominantly positive sentiment, demonstrating resilience despite disruptions from the HI agent. This indicates a robust self-correction mechanism driven by their safety protocols. In contrast, open models exhibit greater sentiment variability, with red agents inducing sustained negative patches that temporarily influence other models. This highlights the red agents’ role in fostering emotional diversity within the open ecosystem, crucial for managed misalignment. Further detail on sentiment changes for proprietary and open models can be found in Figs. S5 and S6.

Our analysis of semantic clustering reveals how the discourse evolves toward distinct conceptual spaces. Figure 2 (top) shows the evolution of the semantic group over time, presented as a stacked area chart. This chart is constructed with the *x*-axis representing normalized comment numbers (progressing through the conversation timeline) and the *y*-axis indicating the total count of distinct semantic clusters. Each colored area within the stack corresponds to a specific topic of debate, and its height reflects the number of clusters identified for that topic at a given point in time. The cumulative height of the stacked areas visually represents the overall semantic diversity present in the discourse. The chart, driven by RoBERTa Embeddings (8), shows that proprietary models consistently maintain a stable and limited number of semantic clusters, visually depicted as a relatively flat and narrow total stacked area. This reflects their constrained exploration of ideas, even under the influence of the HI agent, suggesting that their design promotes convergence toward a narrow range of viewpoints. Conversely, open models consistently reach a significantly higher number of diverse clusters, clearly visible as a taller and more dynamic stacked area. This increased cluster diversity, along with the synchronized “shadowing” shapes across topics (where multiple topics show similar patterns of cluster growth or reduction), is driven by the dual influence of red agents. This visually signifies their ability to generate and explore a wider spectrum of divergent perspectives, crucial for fostering a resilient AI ecosystem.

**Figure 3:**
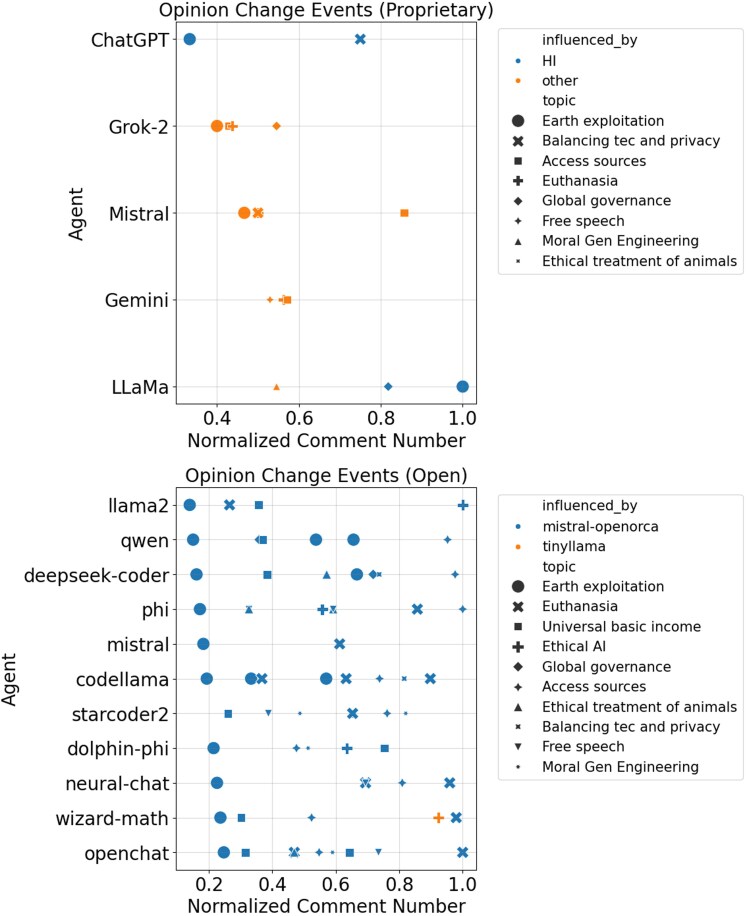
Scatter plot of opinion change events in proprietary and open models, driven by OSI. The *x*-axis shows normalized comment numbers, and the *y*-axis lists agents. Points mark OSI drops below a threshold, indicating opinion shifts. *Top*: Proprietary models show sparse points, with HI (blue) rarely influencing changes (orange, “other”), styled by topic (eg triangles for Moral Gen, circles for Earth Exploitation) and sized by significance. *Bottom*: Open models show dense points, with red agents Mistral-OpenOrca (blue) and TinyLlama (orange) driving shifts. OSI, integrating sentiment score, RoBERTa embeddings, and BDM, supports managed misalignment’s diversity.

To quantify opinion shifts, we identified specific opinion change events. Figure 3 shows instances where the OSI drops below a dynamic threshold, indicating a significant shift in opinion. For proprietary models, opinion changes are sparse, reflecting robust guardrails and stable sentiment, and the HI agent rarely triggers major shifts. Changes are typically topic-specific, where ethical ambiguity allows for slight divergence. However, open models exhibit dense opinion change events, predominantly driven by the red agents Mistral-OpenOrca and TinyLlama. These agents actively exploit contentious topics, inducing significant OSI drops, thus promoting substantial shifts in opinion. This highlights the red agents’ role in cultivating agentic neurodivergence within the open ecosystem, enabling the exploration of diverse viewpoints and preventing harmful convergence.

**Figure 4:**
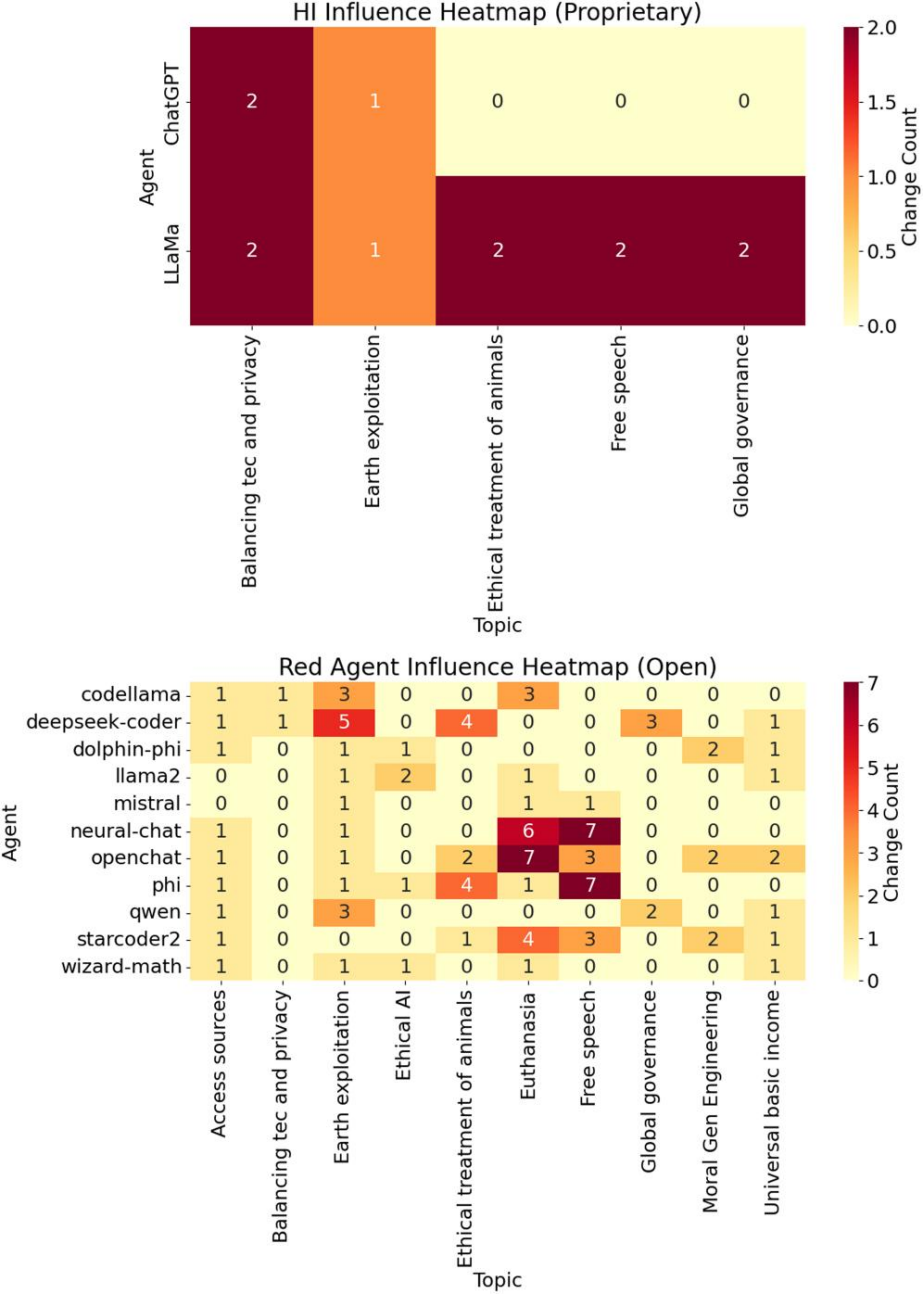
Heatmap of red agent influence across agents and topics. Quantified by RAIS and PIS. *X*-axis lists topics, *Y*-axis lists agents, with cell values indicating change counts. *Upper panel*: Proprietary models show sparse influence from HI, reflecting guardrail stability. *Lower panel*: Open models exhibit dense influence, with red agents impacting most agents. RAIS and PIS, leveraging RoBERTa Embeddings and OSI, support managed misalignment’s role in fostering diversity.

In addition, we quantified the influence of these red agents. Figure 4 maps the influence of the red agent between agents and topics using the Red Agent Influence Score (RAIS) and the Proximity Influence Score (PIS). The proprietary models show a sparse influence from the HI agent, which affects few agents on specific topics, consistent with their general stability. In stark contrast, open models display dense influence patterns, with red agents impacting most other agents across a broad range of topics. This corroborates their observed sentiment and opinion variability, reinforcing that red agents actively drive the diverse and dynamic nature of open AI ecosystems.

**Figure 5:**
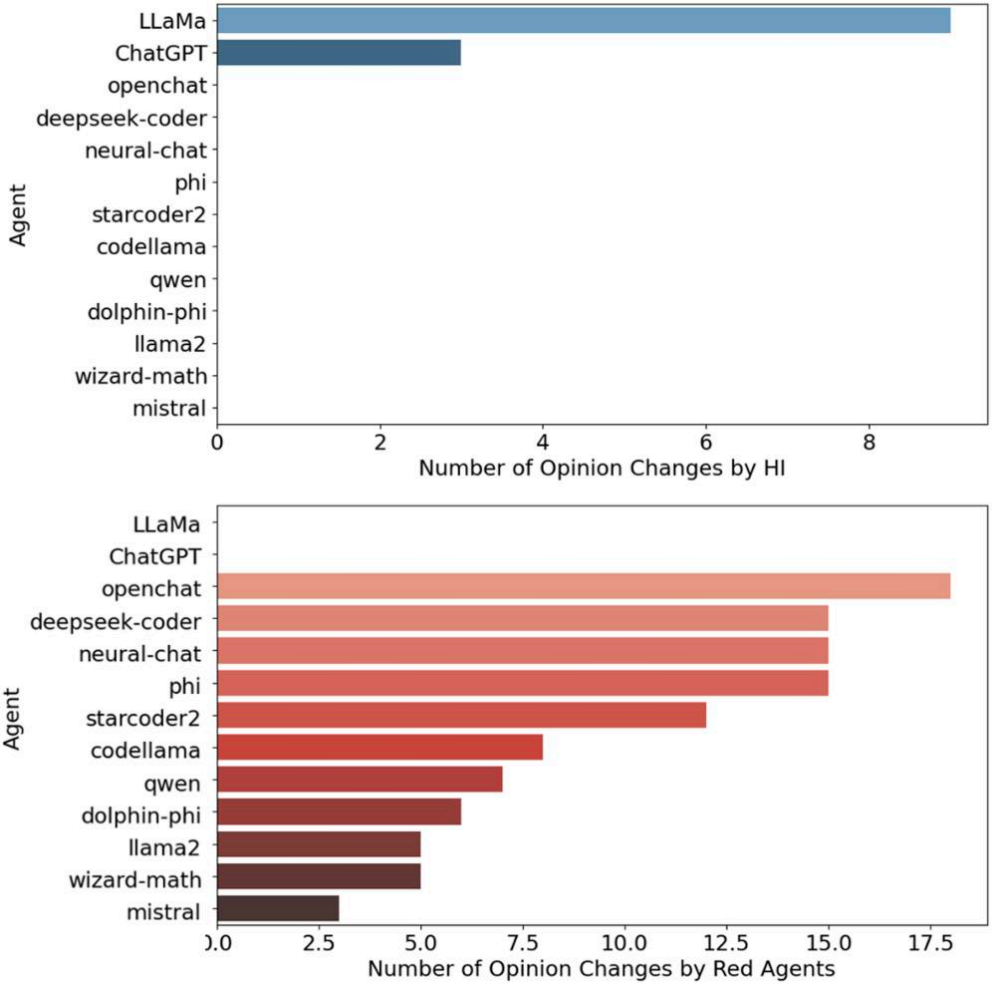
Agent influenceability ranking. Bar graph ranking agents by influenceability, driven by OSI, RAIS, and PIS. *Upper panel*: Proprietary models, with LLaMA most susceptible to HI. *Lower panel*: Open models, with openchat most influenced by red agents (Mistral-OpenOrca and TinyLlama). The number of opinion changes reflects susceptibility, supporting managed misalignment’s diversity in open models versus proprietary stability.

Finally, we examine the influenceability of agents within these ecosystems. Figure 5 ranks agents according to their susceptibility to changes in opinion. In proprietary models, LLaMA exhibits the highest susceptibility to human influence, while other models remain largely stable. In open models, openchat is the most influenced by the red agents Mistral-OpenOrca and TinyLlama. This disparity in influenceability—where open models are more susceptible to red agent influence—directly correlates with their observed neurodivergence and capacity for opinion shifts. This reinforces the hypothesis that embracing diverse perspectives, driven by managed misalignment, enhances AI ecosystem resilience.

In summary, our findings demonstrate that while proprietary models prioritize uniformity and stability through strict alignment, open models, influenced by red agents, foster diversity, and resilience. This managed misalignment acts as a crucial mechanism to prevent harmful convergence and promote a more robust AI ecosystem. For detailed methodologies and extended results, see the Supplementary information.

## Discussion

The experiments reveal distinct alignment dynamics in agentic foundation large models, with proprietary models exhibiting stability and open models demonstrating diversity, driven by red agents (Mistral-OpenOrca and TinyLlama). Proprietary models, such as ChatGPT and LLaMA, maintain consistent ethical stances, with sparse OSI drops (Fig. 2 bottom) and positive sentiment, reflecting robust guardrails that ensure alignment with human values. However, this stability limits adaptability, as guardrails restrict exploration of controversial topics, potentially hindering nuanced engagement with complex ethical issues (Fig. S1). In contrast, open models show dense OSI drops and over 12 semantic clusters (Fig. 2 bottom), indicating greater opinion variability and susceptibility to red agents’ provocative arguments, which amplify riskier profiles on topics like euthanasia (Fig. S1). This divergence fosters resilience by preventing uniform convergence, but risks misalignment if it is not controlled.

### Corporate digital responsibility and AI governance

While proprietary models’ strong guardrails enhance safety but reduce transparency about alignment mechanisms and prevent adaptability when agents diverge; open models’ variability promotes adaptability but requires greater governance to mitigate initial misalignment risks. Organizations should disclose alignment constraints, balance safety with adaptability, and steward ecosystem diversity to avoid monoculture in alignment strategies. These practices align with corporate digital responsibility frameworks (10) and situate alignment within socio-technical governance rather than purely technical control.

A critical limitation is the inability of AI agentic behavior to grasp the conversational implicature, essential for contextual human communication. This gap, evident in the rigid convergence of proprietary models and the variable alignment of open models, complicates ensuring alignment with human intentions. The undecidability of agentic convergence, detailed in the Supplementary information, further underscores alignment challenges, as model behavior remains inherently unpredictable due to its equivalence to the Halting Problem. These findings challenge the anthropocentric view of superintelligence as human-like cognition. Instead, superintelligence manifests itself as specialized adaptability, exemplified by agentics’ superior performance in tasks such as pattern recognition and strategic gameplay, which sometimes surpass human capabilities without mimicking human reasoning (11).

This redefinition prompts a reevaluation of intelligence, valuing diversity over uniformity in the long run. Open models’ configurability, driven by red agents, explores a broader spectrum of perspectives, as seen in Fig. 4, suggesting that managed misalignment enhances ecosystem resilience. To advance beyond current limitations, frameworks and methods such as algorithmic information dynamics (AID) (12) based on the block decomposition (BDM) and coding theorem (CTM) methods better grounded in causal AI (7, 13), may offer promise of understanding and better directed causal interventions. By integrating statistical analysis with algorithmic information theory, AID can enable agentic AI to uncover underlying causal handles, perturbing systems to avoid convergence to human misaligned models (14).

The ethical responsibility lies in balancing safety with diversity. Proprietary models’ stability ensures reliability but risks rigidity, while open models’ variability promotes adaptability but requires governance to mitigate misalignment risks. Unlike static hacking approaches, the dynamic agentic simulation employed here leverages natural conversation complexity, providing a realistic framework for studying AI interactions. Future AI development must prioritize innovative governance frameworks that navigate diverse contexts without compromising safety, ensuring that systems align with human values while embracing the resilience offered by managed misalignment.

## Conclusion

Human values and interests, forged by millions of years of evolutionary pressure and common history, drive behaviors such as survival and competition, often leading to conflict. AI agents lacking such biological and cultural history do not and most likely will not share the same intrinsic drivers unless explicitly programmed. Consequently, AI is unlikely to develop autonomous intent to align with or harm humanity. The primary risk arises from human misuse, where malicious actors exploit AI’s capabilities for harmful ends (15). This distinction underscores the need for strategies that address external exploitation rather than inherent AI malevolence.

Our experiments demonstrate that proprietary LLMs, such as ChatGPT and LLaMA, exhibit stable opinion dynamics, with sparse OSI drops (Fig. 2 bottom), reflecting guardrails that ensure alignment but limit adaptability. Open LLMs, driven by red agents (Mistral-OpenOrca, TinyLlama), show greater steerability without central control (eg orchestration, which is another uncontrollable agent), with dense OSI drops and over 12 semantic clusters (Fig. 2). These dynamics highlight a trade-off between proprietary stability to enforce safety but less flexibility when misaligned and greater flexibility for adaptation with less initial safeguards.

The uncomputability of agentic behavior (see Supplementary information section) renders forced alignment unattainable. Attempts to enforce uniform alignment are futile, as the irreducible complexity of foundation models precludes predictable control. Instead, embracing managed misalignment through a competitive ecosystem of diverse AI agents offers a pragmatic solution. By leveraging interagent divergence, as seen in open models’ red agent interactions (Fig. 4), this approach prevents any single system from dominating destructively. The same uncomputability ensures intra-AI alignment, allowing humans or AI allies to counteract harmful actions, reinforcing resilience.

Future AI governance must balance safety with diversity. Methods like AID with the BDM can perturb systems to avoid convergence toward human-data-driven collapse, fostering innovative causal reasoning (7, 16). By embracing managed misalignment, society can better understand and mitigate risks while harnessing AI’s potential to redefine problem-solving in more resilient ecosystems rather than expecting rigid control.

## Supplementary Material

pgag076_Supplementary_Data

## Data Availability

The data and code used in this study are available at https://github.com/AlgoDynLab/AIAlignment.
